# “I’ll take them another day”: A qualitative study exploring the socio-behavioral complexities of childhood vaccination in urban poor settlements

**DOI:** 10.1371/journal.pone.0303215

**Published:** 2024-05-13

**Authors:** Judy Gichuki, Ben Ngoye, Francis Wafula

**Affiliations:** Institute of Healthcare Management, Strathmore University Business School, Nairobi, Kenya; Zagazig University Faculty of Human Medicine, EGYPT

## Abstract

Despite improvement over recent decades, childhood vaccination uptake remains a concern across countries. The World Health Organization observed that over 25 million children missed out on one or more vaccines in 2021, with urban poor and other marginalized groups being the most affected. Given the higher risk of disease transmission and vaccine-preventable diseases (VPD) outbreaks across densely populated urban slums, identifying effective interventions to improve childhood vaccination in this vulnerable population is crucial. This study explored the behavioral and social factors influencing childhood vaccination uptake in urban informal settlements in Nairobi, Kenya. A grounded theory approach was employed to develop a theoretical account of the socio-behavioral determinants of childhood vaccination. Five focus group discussions (FGDs) were conducted with purposively sampled caregivers of children under five years of age residing in informal settlements. The Theory of Planned Behavior guided the structuring of the FGD questions. An iterative process was used to analyze and identify emerging themes. Thirty-nine caregivers (median age 29 years) participated in the FGDs. From the analysis, four main thematic categories were derived. These included attitude factors such as perceived vaccine benefits, cultural beliefs, and emotional factors including parental love. Additionally, subjective norms, like fear of social judgment, and perceived behavioral control factors, such as self-control and gender-based influences, were identified. Furthermore, a number of practical factors, including the cost of vaccines and healthcare providers attitude, also affected the uptake of vaccination. Various social, behavioral, cultural, and contextual factors influence caregiver vaccination decisions in urban poor settings. Community-derived and context-specific approaches that address the complex interaction between socio-behavioral and other contextual factors need to be tested and applied to improve the timely uptake of childhood vaccinations among marginalized populations.

## Introduction

Immunization is recognized as an essential and effective mechanism for controlling communicable diseases. However, childhood vaccination rates have been declining. The World Health Organization (WHO) estimates that 25 million children missed out on one or more vaccination doses in 2021, representing a five-percentage drop in coverage compared to the 2019 achievement [1].

Kenya has made remarkable progress in the rollout of childhood vaccination since the inception of the essential program for immunization in 1980 [2]. Immunization coverage, however, remains sub-optimal. Based on findings from the 2022 Kenya Demographic Health Survey (KDHS), only 55% of children aged 12 to 23 months had received all the vaccinations recommended for their age [3]. Vaccinations given within the first six weeks after birth have notably higher coverage. For instance, there was an eight-percentage difference in the completion rates of the diphtheria-pertussis- tetanus (DPT) vaccine between the first (97%) and the third dose (89%) [3].

Sub-national data analysis from the demographic survey also revealed variations in vaccination coverage across regions and marginalized groups. As per the national immunization schedule, the percentage of fully vaccinated children in Nairobi stood at only 45%, falling below the national average of 55%. Furthermore, full vaccination coverage was higher in the highest wealth quintile (59%) as compared to the lowest quintile (42%), while 3.5% of children in the lowest quintile had received no vaccinations, in comparison to 1.4% in the highest quintile [3].

Marginalized populations, such as those in urban slums, remote rural areas, or war-prone areas, are at higher risk of being unvaccinated [4, 5]. Additionally, one in five unvaccinated children under one year of age is estimated to reside in urban poor settlements [6]. A longitudinal study in two urban informal settlements in Nairobi reported on-time vaccination coverage below 50% [4]. The Immunization Agenda 2030 recognizes the urgency of reaching the most marginalized populations and integrating successful strategies for immunization delivery [7]. Given the higher risk of disease transmission and vaccine-preventable diseases (VPD) outbreaks across densely populated urban slums, identifying effective interventions to improve childhood vaccination in this vulnerable population is crucial [8].

A range of interventions, including reminders, health education, and motivational interviewing, have been found to be effective in promoting childhood vaccination uptake [9–11]. For instance, pre-and post-natal vaccination education can positively influence parental attitudes and beliefs about infant vaccination [11]. Intervention efforts for marginalized populations have majorly addressed supply-side barriers by improving accessibility and commodity management [12]. Some of the tested interventions in Kenya, including intensified fixed-point immunization services, door-to-door defaulter tracing by community health volunteers, and household-level immunization, have significantly boosted immunization coverage [12]. A combination of mobile phone text message reminders and incentives was also found to significantly improve immunization coverage and timeliness in a cluster-randomized controlled trial in Western Kenya [13]. A multidimensional perspective considering demand and supply-side barriers is pivotal in reversing the declining childhood vaccination uptake [8]. Exploring the social and behavioral dynamics that influence the demand for childhood vaccination facilitates the development of tailored interventions that consider cultural norms and community dynamics. Such interventions are more likely to be accepted and embraced by communities [14].

The WHO Behavioral and Social Drivers (BeSD) of vaccination conceptual model identifies four key domains influencing vaccination uptake. These include caregiver thoughts and feelings on vaccination (including risk perceptions and vaccine confidence), social processes like social norms and recommendations, vaccination intentions, and practical factors around seeking vaccination [15]. Public health programs must consider behavioral and social biases and address context-specific concerns to improve vaccination rates and reduce the burden of preventable diseases.

Applying behavioral sciences in exploring vaccination decision-making processes can help identify health system gaps contributing to under or untimely vaccination. Moreover, individual decisions on vaccination are influenced by how the vaccination choices are presented and how the actual decisions are made [16, 17]. Recognizing the role of beliefs and biases in vaccination decisions complements traditional supply-side approaches toward improving vaccination uptake [16]. In addition, understanding community-derived and context-specific behavioral preferences is vital in overcoming vaccine demand challenges and providing more client-centered care [18].

This study employed the Theory of Planned Behavior (TPB) as an initial conceptual framework to explore the socio-behavioral determinants of childhood vaccination in urban poor populations. TPB, a cognitive framework, encompasses three fundamental constructs: attitude (the individual’s positive or negative evaluation or appraisal of the behavior), subjective norms (perceived social pressure that encourages or discourages the behavior), and behavioral control (perception of how easy or challenging it is to perform the behavior) [19]. The constructs are posited to influence an individual’s intention to engage in a specific behavior, consequently affecting their actual behavioral performance. Previous research has demonstrated the significant predictive power of TPB constructs in relation to vaccination intentions [20, 21]. Kim and Choi (2016) found a 69.5% explanatory power for subjective norms, attitudes, and perceived behavioral control in predicting vaccination intentions [21]. By exploring how attitudes, subjective norms, and perceived behavioral control intersect to shape childhood vaccination, we aimed to identify opportunities tailored to address the unique needs and challenges in urban informal settlement populations. We also sought to expand on TPB constructs to identify emerging factors influencing vaccination uptake in urban poor settlements. This comprehensive approach facilitated an intricate understanding of the complexities surrounding sub-optimal vaccination within urban poor settlements. Consequently, this study explored the socio-behavioral factors contributing to sub-optimal childhood vaccination in the urban poor settlements of Nairobi, Kenya.

## Methodology

### Study design

This qualitative study employed a grounded theory approach to develop a theoretical account of the socio-behavioral determinants of childhood vaccination grounded in data from the participants’ perspectives and experiences [22]. Study methods and results are reported following the Standards for Reporting Qualitative Research (SRQR) [23].

### Study setting

This study was conducted in the urban poor settlements of Nairobi, the capital city of Kenya. Based on data from the 2019 nationwide census, Nairobi is the most populous county in Kenya, home to approximately 4,397,073 inhabitants [24]. Nairobi has a cosmopolitan urban population, with over 60% estimated to reside in informal settlements [25]. The informal settlements comprise approximately 5% of the total residential area and are characterized by highly dense residential spaces marked by overcrowding, limited access to essential services, and inadequate infrastructure [25].

Children from poorer backgrounds in Nairobi are less likely to be fully vaccinated, yet they represent the most vulnerable population based on their living conditions and caregiver capacity [4, 26].

### Study population and eligibility criteria

The study population consisted of parents of children below five years of age. Participants were eligible if they were 18 years of age or above, had a child below five years of age, were residents of an informal settlement in Nairobi, and if they gave consent to participate in the study. Participants who did not provide consent were excluded from the study.

### Sampling and recruitment

Focus group discussions (FGDs) were held with parents of children below five years of age. Purposive theoretical sampling, which focused on theoretical relevance and purpose, was employed to recruit participants. In theoretical sampling, data collection is shaped by insights gained from continuous FGD data analysis [27]. This iterative process utilized emerging theoretical concepts to guide further data collection. For instance, as gender-based concerns emerged as a crucial element influencing caregivers’ vaccination behavior, a subsequent focus group discussion was conducted with male participants to enhance comprehension of the matter.

Participants were recruited from four purposively selected informal settlements in Nairobi. Community health volunteers (CHVs) attached to households in the four purposively selected informal settlements assisted in identifying participants. The research team guided the CHVs regarding recruitment objectives and eligibility criteria. The purposive selection aimed at capturing a range of perspectives and experiences related to vaccination behaviors within the study population. This included caregivers whose children were up to date with vaccination and those who had delayed their children’s vaccination. The recruitment process was initiated in the week prior to each FGD.

Each FGD involved face-to-face discussions with groups of seven to nine caregivers. Due to the cultural and societal gender dynamics, female and male FGDs were run separately to bring out unique experiences and perspectives based on gender roles and societal expectations.

Five FGDs were conducted between 3^rd^ October and 1^st^ December 2022, each lasting an average of 90 to 120 minutes. Theoretical saturation served as the criterion for determining the completion of the data collection process. This was defined as when no new information or data emerged from additional data collection and analysis [22].

### Data collection

The principal investigator moderated the FGDs while a research assistant with extensive knowledge of primary healthcare took notes. Both had experience in conducting FGDs and were fluent in Swahili and English. To ensure a comfortable and conducive environment for participants, a same-gender community health assistant was present during each FGD to assist with logistical aspects and to provide support as needed.

The discussions were conducted in community social halls to enhance the participants’ freedom to communicate issues. The community social halls were within walking distance for participants. Audio recordings supported by field notes were used to document the data collection process. Participants’ permission to audio record the FGD sessions was sought before the recording. The FGDs were conducted in Swahili to facilitate active participation and effective engagement with the participants.

A semi-structured, open-ended topic guide that allowed participants to share their thoughts, experiences, and perspectives was utilized. Active listening and probing techniques encouraged participants to elaborate and engage in dialogue. The structuring of the topic guide was based on the Theory of Planned Behavior (TPB) [19, 28]. Selection of the TPB was guided by its applicability in providing insights into the factors that shape different health behaviors [29]. The FGD topic guide is provided in supplementary material (S1 File).

The FGDs explored caregivers’ attitudes toward vaccination. They examined both the instrumental (caregivers’ beliefs about vaccination outcomes) and affective attitude components (caregivers’ feelings and emotional reactions associated with vaccinating their children) [30]. Injunctive norms (behavior that individuals participate in because they perceive that others expect it) and descriptive norms (behavior that individuals adopt because they believe that others in their reference group also engage in the same behavior) [14] were also explored. FGDs also examined perceived behavioral control factors and emerging socio-behavioral themes. Anonymous demographic data, including caregiver’s age, youngest child’s age, total number of children, and education level, wascollected prior to the FGDs to provide context on participant diversity.

### Data analysis

Audio recordings were transcribed verbatim in Swahili, translated to English, and back-translated to Swahili to ensure no loss in meaning before being transferred into QDA Miner Lite software [version 2.0.9]. All audio recordings and transcripts were stored securely on password-protected computers, only accessible to the research team.

An iterative approach guided by the tenets of grounded theory was used in the analysis, starting with an initial coding framework, ongoing refinement, and analysis of themes as new insights and feedback emerged from the interview data [22]. Two authors worked independently to code each transcript and then reviewed the coding together for alignment. This was followed by examining the codes to identify recurring concepts and connections for each transcript. Similar codes were then grouped into categories and subcategories. These were subsequently charted, and mapping and interpretation were conducted to identify and discuss important themes, patterns, and observations.

A team of three researchers conducted the study: the PI, a researcher with expertise in immunization programs, and two co-investigators with extensive experience in conducting health service research. An audit trail was maintained throughout the research to enhance the study’s trustworthiness and rigor. This included detailed documentation of participant recruitment, data collection, and data analysis processes. The research team was aware of the potential impact of their personal and professional backgrounds on the research process and engaged in ongoing reflexivity throughout the study. The team openly discussed their assumptions and critically reflected on their potential impact on the research process and findings. The team also sought feedback from peer reviewers and CHVs who participated in the study.

### Ethical considerations

Ethical approval was obtained from the Strathmore University Institutional Scientific and Ethical Review Committee (SU-IERC) and the National Commission for Science, Technology and Innovation (NACOSTI). Informed written consent was obtained from all individual participants, and confidentiality of the information provided by the participants was ensured.

## Findings

A total of 39 caregivers, including both mothers and fathers of children under five years old, participated in the FGDs. The median age for the participants was 29 years (range 20–52). Most of the participants had completed secondary education (54%), while a fifth were male. See Table 1 for socio-demographic characteristics.

**Table 1:** Socio-demographic characteristics of participants.

Demographic characteristic (n = 39)	
Age (median, range), years	29 (20–52)
Age of youngest child (median, range), months	24 (3–48)
Number of children (median, range)	2 (1–6)
Sex	
Female	31 (79.5%)
Male	8 (20.5%)
Education level	
Primary	7 (17.9%)
Secondary	21(53.8%)
College	4(10.2%)
Missing	7(17.9%)

The results are presented by themes, subthemes, and selected representative quotes and are summarized in four major themes (Table 2). The sub-categories,codes and qoutes are provided in S1 Table.

**Table 2:** Summary of emerging themes.

Theme	Sub-themes
Attitude	Role of knowledge and beliefs
	Role of emotional factors
Subjective norms	Role of family, friends, and peers
	Fear of social judgment
Perceived Behavioral control factors	Self-control factors
	Past behavior
	Gender-based factors
Practical factors	Ease of access to health services and healthcare providers’ attitude

### Theme 1: Attitude

Participants’ vaccination knowledge and beliefs influenced their attitudes toward vaccination.

#### Sub-theme I: Role of knowledge and beliefs

All interviewees appeared to have basic knowledge of the benefits of vaccination in disease prevention. Other benefits mentioned included making the child stronger and healthier, preventing infections from other children, and preventing paralysis or disability. Some participants also stated the advantage of avoiding additional medical costs.

“*And*, *if they are vaccinated*, *our cost of going to pay at the hospital will not be there*. *But when the baby isn’t vaccinated*, *when they start having those problems*, *it starts eating up your pocket money and time*.*” (Male FGD)*

Vaccines were perceived to be effective because children did not contract diseases during outbreaks, or they did not get severe illnesses if they were vaccinated.

“*It works because even if your child gets caught with that chicken pox*, *they are not defeated; two days they recover*.*” (Female FGD)*

Most participants indicated that they knew about some vaccine side effects, including pain, excessive crying, swelling in the injection site, diarrhea, fever, and vomiting. The side effects were perceived to depend on how the vaccines had been stored and the healthcare provider’s experience in giving injections. Mistrust in the quality of vaccines was also highlighted as a contributing factor to the failure to take children for vaccination and represented community perspectives rather than individual participants’ experiences. For example, an FGD participant reported that a few women did not bring children to be vaccinated because they believed the vials contained water or expired vaccines. A few caregivers also talked of having mistrust in the vaccination process where they felt the healthcare provider was not sufficiently competent, citing poor injection techniques that pose the risk of disability.

“*You get like those vaccines for the legs …you get I don’t know if the child was injected badly…*. *they start reporting that the child has never kept quiet after being injected with that vaccine*. *Then you get that the leg starts to develop problems*, *you start to wonder if it’s that vaccine that was injected badly or that drug got stuck there*, *so also another effect that has been in several children*.*” (Male FGD)*

Religious beliefs were also seen to influence the uptake of vaccines in all the FGDs. Caregivers in some religious formations did not immunize their children because they were forbidden or believed in protection through prayer. Cultural beliefs also contributed to vaccination delays. For instance, there was fear of adverse effects of injections where a child had been looked at with "evil eyes" or "thrown for things" (depictions of witchcraft). This was interpreted through specific observations, such as severe illness that did not resolve through conventional medicine, swelling of gums, and swollen bellies. Some caregivers feared that injections could cause death in such cases. Such beliefs were perceived to vary based on tribe, customs, and knowledge.

“*When these things come out*, *you can’t take them*. *Because if that needle tries to be given to them*, *your baby*, *that’s how you forget them*. *Just like that*, *just any needle*, *as long as they receive it in their body*, *that’s how they are gone*. *These are not things that we should play with*, *we have to be careful with them*.*” (Female FGD)*.

#### Sub-theme II: Role of emotional factors

Parental love was highlighted as a powerful positive attitude that promotes vaccination and motivates caregivers to vaccinate their children to protect them from illness.

“*This is because I love my child so much*, *I love my family life*, *I don’t want them to get these diseases; our world has changed with different diseases*.*” (Female FGD)*.

Fear and anxiety about vaccination were perceived to arise from inadequate information about vaccines.

“*So those are the things that happen*, *they scare someone*, *the baby has been vaccinated almost twice a month*, *you see*, *so one wonders what this other vaccine is for*, *now that fear is getting into them*.*” (Female FGD)*.

Anticipated regret from failure to vaccinate the child also influenced caregiver vaccination decisions. Some participants stated that they would feel bad or regret if their child developed a disease such as measles due to their failure to take the child for vaccination.

“*I can blame myself because the mistake is mine*.*” (Female FGD)*.

“They’ll feel really bad, they will regret it.” (Female FGD).

### Theme 2: Subjective norms

Different subjective norms influenced caregivers’ decisions on vaccinating their children.

#### Sub-theme I: Role of family, friends, and peers

Family members, friends, and neighbors were influential in vaccination decisions. Various injunctive and descriptive norms were mentioned, including influence from partners, family members, and other social circles.

“*My grandmother told me its not a must*. *That long ago they weren’t getting vaccinated*, *also those of mine I shouldn’t take*, *but now you take them voluntarily*.*” (Female FGD)*.

“*A friend of mine told me it wasn’t necessary*, *because previously they were only giving up to nine months…………*: *Because they didn’t even take their own*.*” (Female FGD)*.

The influence from family and friends was perceived to be due to a combination of factors, including divergent views within their community and inadequate information on the necessity of additional vaccinations. A few participants pointed to their peer’s disapproval of mass vaccination campaigns that influenced their decisions to vaccinate their children.

“*Now you see others are heartbroken because people are saying if mine was vaccinated last time*, *this one that has come again*, *it is for what*? *But now you get if it can be explained to you*, *you’ll find it’s of importance*, *it’s because you haven’t been told that you listen to someone telling you not to take them*.*” (Female FGD)*

On the other hand, some participants reported ignoring negative advice from their social networks. Decisions to disregard negative comments from peers were based on various factors, including personal beliefs and access to accurate information. A participant stated:

“*Now if you know yourself*, *you can’t listen to them when they say the vaccines come with deformities*.*”* (Female FGD)

#### Sub-theme II: Fear of social judgment and stigma

The fear of social judgment and self-stigma were key barriers to vaccination. Caregivers’ concerns about being judged by peers and providers caused some not to seek vaccination services. This included the fear of being seen as neglectful parents when children were untidy or underweight.

“*When you get there*, *they say you don’t give the baby food*, *like that*, *you see*. *Now someone sees I’m going to go there to meet the doctor*, *mess with my spirits; now you see you should stay home*.*” (Female FGD)*.

Some caregivers feared being socially judged because they lacked the financial means to conform to societal expectations regarding their children’s dressing etiquette—notably, the inability to purchase diapers contributed to suboptimal vaccination.

“*You don’t have money for pampers*, *and you see where you take them to the hospital when you go*, *it’s obvious you’ll be told*, *if they are being weighed kilos*, *to remove their clothes*, *now don’t you see there’s that shame……*. *now you see that shame*, *you say*, *let it pass just so*.*” (Female FGD)*.

One participant also mentioned caregivers’ fear of being stigmatized due to their child’s HIV (positive) status as contributing to missed vaccinations.

“*I’ve given birth to a baby*, *and they are positive; there’s one who finds it difficult to take the baby to the clinic because now she sees when I get there*, *everybody*, *the doctor will know I’m like this*, *this one will know I’m like this*, *now you see*.*” (Female FGD)*.

### Theme 3: Perceived behavioral control factors

Caregivers’ self-control, as well as their past experience, affected the timely uptake of vaccination.

#### Sub-theme I: Self-control

Caregivers’ self-control was influenced by competing work demands and financial constraints. Work commitments were highlighted as a critical factor affecting the timeliness of vaccinations.

“*Today it’s a clinic day*, *and then someone tells you I have some work you can do for me*, *and you have nothing in the house*, *you say*, *let me do work first; I’ll take them (to the clinic) another day*.*” (Female FGD)*.

Self-control also depended on the ability to carry out the necessary preparations before the vaccination visit, such as ensuring that household chores were performed before the vaccination visits.

#### Sub-theme II: Past experience

Past experience with vaccination contributed to caregiver vaccination decisions. Some participants expressed confidence in childhood vaccines because they had been used for generations and desired their children to experience the same benefits.

“*I believe the vaccine is from long ago because it is not coming today*. *I believe because we got those vaccines and our kids*, *we would want them to grow like us who got those vaccines*.*” (Female FGD)*

At the same time, some noted complacency among some caregivers in their community who did not see the value of vaccines, arguing that the older generations never got vaccinated, yet they lived to an old age.

“*People say that in the past*, *our parents weren’t getting vaccinated*, *and they were surviving*. *They don’t see the need to take children*. *You get a mother; even if you tell her you’re taking the baby up to five years old*, *she tells you that when she reaches nine months*, *she cannot continue*.*” (Female FGD)*

Direct or indirect negative experiences with previous vaccinations contributed to fear of subsequent vaccinations.

“*Some say those vaccines*, *like this one for polio*, *sometimes you hear that after the vaccine*, *someone is disabled after they got that vaccine*, *you find others are afraid to give it to children not to get it (disability)*.*” (Female FGD)*.

#### Sub-theme III: Gender-based factors

In all the FGDs, a child’s vaccination was perceived as the mother’s responsibility as she was the primary caregiver and would bear the consequences if the child developed a disease.

“*Mostly*, *you know*, *we don’t know what is written in those books*, *the mother knows what is written*. *Sometimes if you are at work*, *you hear her telling you she should attend the clinic*, *now those books*, *she knows how they’ve been written*, *and she knows how she is going to the clinic*.*” (Male FGD)*

In the female focus group discussions, caregivers expressed varying levels of support from their spouses in the vaccination process. Some received support from their spouses, who accompanied them to the clinic. In contrast, others felt their spouses were occupied with work, and the vaccination responsibility solely rested with the mother.

“*Now I am the one volunteering to do the job because he has gone to search (for work); if we both go*, *what will we eat*?*” (Female FGD)*

Most male participants stated that their role in the vaccination process was mainly supportive, reminding the mothers about the vaccination dates and providing financial support for transport costs and clinic fees. However, some participants felt that vaccination decisions should be a combined effort, as the mother may not always be there to take the child to the clinic or may forget.

Although mothers were responsible for deciding whether children should receive vaccines, their ability to choose was limited by a lack of autonomy on financial matters in the household. Where the clinic was far, and transport was required, or where the mother had to go to a private clinic, the decision to vaccinate was contingent on the financial capacity and willingness of the spouse.

“*That day you have told him he’ll tell you yes*, *but when that date comes*, *maybe where he was thinking he’s going to get it (money)*, *he goes and misses*, *what is he going to do*, *they will tell you to take them some other day*.*” (Female FGD)*

Three female FGDs identified marital conflicts as contributing to delayed childhood vaccination. Marital disputes were perceived to emanate from the difficult living conditions in the informal settlements. When the child’s vaccination appointment coincided with the marital disputes, various factors, including the mother deserting the family home or not receiving financial support, such as transportation to the clinic from their partner, led to failure to take the child for vaccination. In cases where disputes escalated into violence, the resulting physical scars resulted in feelings of shame and stigma, deterring mothers from taking their children for vaccination on the scheduled date.

“*Oh*, *now we’ve broken up with my husband*, *at night we’ve argued with each other*, *today it’s the baby’s clinic day*, *I’ve escaped*, *that day I can’t take them to the clinic*, *how will I take them*, *and maybe I have been beaten in the house*, *I have marks*, *how are you going to go to the hospital when you’re like that*?*” (Female FGD)*

### Theme 4: Practical factors

Challenges associated with accessing vaccination and healthcare provider-related factors influenced timely vaccination uptake.

#### Sub-theme I: Accessibility, affordability, and ease of access

Poor access to public facilities in informal settlements and the need for transport funds led caregivers to seek vaccination from nearby private facilities. However, the high cost of immunization at the private facilities served as a deterrent.

“*Yes*, *sometimes*, *I go to that private hospital; if it is the starting one*, *you are told 150*, *now some days when you return you are told 50*, *30 you see*, *sometimes you can delay the baby because you don’t have that money*.*” (Female FGD)*.

Delayed vaccination was partly attributed to the inconvenience caused by limited opening hours at public facilities, such as not operating on weekends. Missed vaccination was also attributed to caregivers’ loss of the mother-child booklet due to factors such as house fires. Some caregivers reported that they could not receive vaccination services without the booklet.

“*Now that clinic book is everything; if there is none*, *you will not be taken care of the baby*.*” (Female FGD)*.

#### Sub-theme II: Healthcare providers’ attitude

The negative attitude from healthcare providers was also identified as a primary reason for missed vaccinations. Mothers whose children had missed previous vaccination appointments felt mistreated when they finally presented to the health facilities.

“*Insults or you are put aside when others are being served*, *and your card is put down*, *and the mountain (of cards) is there*. *Like me*, *there was a time I delayed the clinic*, *I was subjected to that contempt*.*” (Female FGD)*.

Some mothers would seek vaccination services at alternative health facilities out of fear of facing repercussions from healthcare providers, while others would endure mistreatment. Some mothers with underweight babies reported hesitating to take their children for vaccination due to the fear of adverse reactions from healthcare providers.

“*Others*, *your child is probably underweight*, *they say you will go and be shouted down by a doctor*, *so you’re afraid*.*” (Female FGD)*.

Similarly, some caregivers did not take their children for vaccination due to the fear of being reprimanded for not spacing out their children through family planning.

“*May be my baby is still young*, *and there I am*, *am pregnant*, *and my child is still small*. *When I get there*, *I’ll start to be quarreled and am told you don’t know family planning*, *and I don’t know what*, *yes*, *I’m going to be afraid now*.*” (Female FGD)*

## Discussion

This study explored the socio-behavioral factors influencing childhood vaccination behavior in urban poor settlements. Utilizing the grounded theory process facilitated concentration on the contextual and process-oriented aspects, along with the choices and behaviors of caregivers about whether they vaccinated their children. Starting with the theory of planned behavior (TPB), we explored the contextual behavioral and social factors that influence timely vaccination uptake in the informal urban settlements of Nairobi. The study findings indicate that perceived vaccine benefits, perceived disease risk, knowledge of vaccine effectiveness, perceived vaccine safety, trust in vaccines, and the vaccination process influence the caregiver’s attitude towards vaccination and their intent to vaccinate their children. Furthermore, cultural, and religious beliefs, social norms, peer influence, and community dynamics also shaped vaccination decisions and actions.

Various extensions of the TPB have been proposed to enhance its explanatory power and to provide a more robust theoretical framework for understanding the complex factors that influence human behavior. For example, the Integrated Behavioral Model (IBM) combines concepts from TPB with other factors, such as environmental constraints and salience of the behavior [28]. The Reasoned Action Approach (RAA), on the other hand, splits the three TPB constructs into pairs of separate yet interconnected sub-components. These include experiential and instrumental attitudes and injunctive and descriptive norms, while perceived control is divided into capacity and autonomy components [29]. Our findings align with the inclusion of instrumental attitude as a subset of TPB’s attitudes and beliefs [19, 29]. Instrumental attitude, in this sense, refers to beliefs about the expected outcomes from a particular behavior [31]. These are distinguished from affective attitudes, which refer to beliefs regarding the likely emotional consequences of behavior [30, 31]. To the latter, our findings suggest that positive and negative affective attitudes and anticipated regret—the adverse emotional reaction resulting from comparing the expected outcome of inaction to the outcome of taking action [32, 33]- may influence vaccination intentions.

Consistent with previous research [34], our findings also point to influences from healthcare providers, family, and friends on the caregivers’ decision to vaccinate their children. This direct influence by others, coupled with the need to conform to social systems and the fear of social judgment and self-stigma, can be aggregated under TPB’s subjective norms–beliefs about the perceived position of important others towards the behavior [19]. However, consistent with the thinking in social psychology, we find it important to distinguish between descriptive norms–the perceived prevalence or typicality of a given behavior, and injunctive norms as the perceived degree of social approval or disapproval of the behavior [35, 36]. Such distinction is essential because, as elaborated by Jacobson and colleagues [36], the descriptive norms provide information relevant to the intra-personal choice of appropriate behavior. At the same time, the injunctive norms include valuable information for guiding, building, and maintaining interpersonal and social relationships. These are, therefore, distinct forms of social influence that can be independently or concurrently targeted by a health promoter keen on understanding, predicting, and influencing health-seeking behavior. Health messaging needs to take cognizance of such social influences and be tailored to the cultural dynamics in the informal settlements.

Gender-related barriers were an important social factor influencing timely childhood vaccination uptake in informal settlements. Though the mothers were identified as the primary decision-makers in vaccinating their children, their male partners played a crucial role in supporting the vaccination process. The lack of financial autonomy among the mothers also greatly influenced their ability to have their children vaccinated on time. Empirical evidence exists. A study analyzing women empowerment and immunization data from demographic and health survey data from fifty countries found that children from mothers with low and medium levels of social independence had three times higher odds of not receiving diphtheria, pertussis, and tetanus (DPT) vaccines as compared to those with high levels of autonomy [37]. Children whose fathers were involved in vaccination decisions and processes were also perceived to be more likely to receive vaccination on time. Indeed, the Immunization Agenda 2030 recognizes the need for tailored strategies to overcome gender-based immunization barriers by increasing male involvement in vaccination through targeted communication to expand their inclusion in vaccination decisions and processes [38]. Involving fathers in decision-making and addressing their concerns about vaccines can help ensure that more children are vaccinated and protected.

The role of information in influencing attitudes, beliefs, and behaviors cannot also be gainsaid. In this study, we found that caregivers still have misconceptions and fears concerning the effects of vaccination that need to be addressed. Most of the misconceptions arise due to inadequate information on vaccination side effects. Other studies have also emphasized the negative impact of insufficient information among caregivers on their vaccination practices [39, 40]. When caregivers lack sufficient understanding, they may be more hesitant or resistant to vaccinating their children, leading to lower vaccination rates. Tailoring messaging to address specific caregivers’ concerns can help to alleviate fears or misconceptions that they may have about vaccines.

The study findings highlight the important role of healthcare providers’ interactions with caregivers in facilitating timely vaccination. Caregiver perceptions of negative treatment by healthcare providers were linked to their vaccination behavior. Similarly, in a study by Braka et al. (2012), participants stated that difficulties in approaching health providers increased their reluctance to communicate their vaccination concerns [39], underscoring the importance of good communication by providers in improving uptake.

Caregivers’ fear of being judged or stigmatized by providers or peers for various reasons also contributed to missed vaccination. This aligns with a study in Uganda that found that the stigmatization of mothers with poorly dressed children by health workers and other women was a barrier [41]. Interventions are needed, particularly in poor urban areas, to minimize missed vaccinations due to caregivers’ fear of being judged or stigmatized by healthcare providers or peers. Such interventions should promote non-judgmental attitudes towards caregivers, including training providers to provide respectful and supportive care to all caregivers regardless of their social status or other factors.

Finally, the respondents raised many practical issues, including accessibility, affordability, and ease of access to health services. Immunization programs need to identify interventions to minimize these missed opportunities, for instance, by using electronic immunization registers (EIRs) as sources of information on children’s vaccination status [42].

That said, our study has some limitations. We used CHVs to gain entry into the informal settlement communities. One potential limitation was the possibility of CHVs omitting eligible participants and favoring individuals they knew. To address this concern, the criteria for participant selection were clearly explained to the CHVs, but it is still possible that some degree of bias may have been present in the sample selection process. Another limitation is the potential difficulty in generalizing the findings due to the focus on urban poor settlements and the small number of participants, typical in qualitative studies. While our findings are based on research conducted in a specific urban poor settlement, they may still have potential applicability in other settings, including other urban informal settlements. Efforts to promote vaccination uptake should consider the unique characteristics in each setting while recognizing the common underlying factors that influence vaccination decisions. Third, during the data collection process, participants were sometimes prompted to discuss the experiences of their peers, which may have made it difficult to determine whether they were speaking about their own experiences or those of others. To address this, quotes were taken within the context of focus group discussions, which served as the unit of analysis.

Nonetheless, this study provides insights into the socio-behavioral factors influencing childhood vaccination in urban poor settlements. The findings allow us to improve and extend the theory regarding socio-behavioral influences. Consistent with the original TPB, intention is the most proximal predictor of behavior, while attitude, subjective norms, and control beliefs are the precursors to intention. However, we propose a clear distinction between instrumental and affective attitudes, and on subjective norms, we distinguish between injunctive and descriptive norms. By addressing these dimensions, interventions can resonate with caregivers’ vaccination outcome considerations, emotions, and connections, fostering greater acceptance and support [30].

## Conclusions

In conclusion, the findings underscore the complex interplay of socio-behavioral factors in caregivers’ vaccination decisions for their children. The findings also support the extension of the TPB to include instrumental and affective attitudes as well as the distinction between injunctive and descriptive norms. These theoretical expansions facilitate a more precise understanding of vaccination decision-making and behavior, informing the development of improved strategies to promote vaccination uptake. The results also emphasize the importance of addressing barriers such as affordability, gender-related factors, information gaps, and healthcare provider attitudes to enhance vaccination uptake effectively.

## Supporting information

S1 Checklist (DOCX)

S1 File https://figshare.com/articles/online_resource/Focus_group_interview_guide_for_caregivers_pdf/22645552. (PDF)

S1 Table FGD sub-categories and codes. (XLSX)
