# Supplementary material for: “I’ll take them another day”: A qualitative study exploring the socio-behavioral complexities of childhood vaccination in urban poor settlements
Source: PLoS One. 2024 May 13;19(5):e0303215. doi: 10.1371/journal.pone.0303215 (PMC11090334; doi:10.1371/journal.pone.0303215)
Supplement: S1 File — (PDF) [file pone.0303215.s002.pdf]

Focus group topic guide for caregivers

**FGD Number**	
**FGD participants**	
**Date /Time**	
**Location**	
**Interviewer identifiers**	

Warm-up

Hello everyone, we are grateful to have all of you here. My name is [INTERVIEWER'S NAME] with [INSTITUTION OR ORGANIZATION NAME]. My friend here will also her/himself.

We are having this discussion with care givers to learn more about your thoughts on the vaccination of your children. We are interested in hearing your opinions. This focus group discussion will be audio-recorded, and a note-taker will be present to document what we are talking about. Your responses will remain confidential, and no names will be included in the final report.

Please note that there are no right or wrong answers to the focus group questions. We want to hear the many varying viewpoints and would like for everyone to contribute their thoughts. Out of respect, please refrain from interrupting others. However, feel free to be honest even when your responses counter those of other group members.

Now we would like to get to also know you. Please tell us your name and how old your youngest child is.

Thank you, now let's start

**PART A – Beliefs, subjective norms, perceived control and biases**	
**Beliefs and attitude about vaccination**	Please take a few minutes to tell us what you feel about childhood vaccination<br>(1) What do you see are the advantages and benefits of your children receiving all the recommended vaccination on time?<br>2) Do you think vaccines work? Why?<br>3) What do you see are the disadvantages and risks of your children receiving all the recommended vaccinations?

**Subjective norms**	(1) Who are the individuals or groups that you talk to about vaccination? Why?<br>(2) Who are the individuals or groups who would disapprove of your child getting all the recommended vaccinations? Why?
**Perceived control factors**	(1) What factors make it easy or enable you to ensure children get all the recommended vaccinations? Why?<br>(2) What are the factors or circumstances that would make it difficult or prevent caregivers from ensuring that children get all the recommended vaccinations? Why?<br>3) Thinking back to the first time you took your child to be vaccinated, tell me how you knew it was time to do so?
**Biases**	1)What do you think is the probability of your children getting a vaccine-preventable disease such as measles?<br>2) How would the thought of your child getting a disease that could have been prevented through vaccination influence if you take them for vaccination? Why?<br>3) How would the thought of your child reacting to vaccines influence if you take them for vaccination?<br>4) What are the factors that would make you do what is more urgent instead of taking your children for vaccination?
**PART B: Perceptions on messaging about childhood vaccination**	
**Messenger**	a) Who are your sources of information about childhood vaccination?<br>b) How much do you trust the different sources of information you have named? Why?<br>c) Which sources do you think are best in influencing you to bring your child for vaccination?
**Approach**	a) Which are the type of messages you have received on immunization (Probe: health education sessions, narrative/stories, pictures, videos, audio-visual advertisements, internet)<br>b) Which of the approaches you have mentioned are most appealing to you?

	c) What, in your opinion, is the best time to receive information about vaccines? Why?
**Message Framing**	Suppose I give you two different messages, message A that says, "if you don't take your child to be vaccinated, they will get sick"; and B that says "if you take your child to be vaccinated, they will be protected and in good health"<br>Which of the two messages do you think is more convincing? Why?

Do you have any additional information you would wish to share about childhood vaccination?

Thank you for your inputs in this discussion.
If you have any other information to share with the interviewers, please feel free to contact us using the provided phone number.
